# Morphological features of single cells enable accurate automated classification of cancer from non-cancer cell lines

**DOI:** 10.1038/s41598-021-03813-8

**Published:** 2021-12-21

**Authors:** Zeynab Mousavikhamene, Daniel J. Sykora, Milan Mrksich, Neda Bagheri

**Affiliations:** 1 Department of Chemical & Biological Engineering, Northwestern University, 2145 Sheridan Road, Evanston, IL 60208 USA; 2 Department of Biomedical Engineering, Northwestern University, 2145 Sheridan Road, Evanston, IL 60208 USA; 3 Departments of Biology and Chemical Engineering, University of Washington, 1410 NE Campus Parkway, Seattle, WA 98195 USA

**Keywords:** Computational biology and bioinformatics, Classification and taxonomy, Machine learning, Cancer imaging, Cellular imaging, Cytoskeleton

## Abstract

Accurate cancer detection and diagnosis is of utmost importance for reliable drug-response prediction. Successful cancer characterization relies on both genetic analysis and histological scans from tumor biopsies. It is known that the cytoskeleton is significantly altered in cancer, as cellular structure dynamically remodels to promote proliferation, migration, and metastasis. We exploited these structural differences with supervised feature extraction methods to introduce an algorithm that could distinguish cancer from non-cancer cells presented in high-resolution, single cell images. In this paper, we successfully identified the features with the most discriminatory power to successfully predict cell type with as few as 100 cells per cell line. This trait overcomes a key barrier of machine learning methodologies: insufficient data. Furthermore, normalizing cell shape via microcontact printing on self-assembled monolayers enabled better discrimination of cell lines with difficult-to-distinguish phenotypes. Classification accuracy remained robust as we tested dissimilar cell lines across various tissue origins, which supports the generalizability of our algorithm.

## Introduction

Cancer has long been the second-leading cause of death in the US^1^. Many cancers—such as breast, liver, thyroid, pancreas, and skin (melanoma)—have continued to rise with the obesity epidemic^2^. Yet, the overall decrease (~ 26%) in American cancer mortality from 1990–2015 demonstrates that healthier lifestyles (e.g. reduced tobacco use) and improved detection capabilities lead to better clinical outcomes^2^. Image classification is a fundamental step in successful cancer detection, and automated technologies have been used to complement expert pathologists^3^. However, the accurate description of cancer remains a significant challenge. Not only is it difficult to obtain enough material to run robust image and genetic analysis, tumor microenvironments (TMEs) also possess heterogeneous phenotypes—especially primary to secondary tumor sites^4^—that potentially obfuscate computer-based analysis. Furthermore, variability in specimen preparation can lead to varying diagnoses, even amongst experts^5,6^. Cancer classification is often empowered by imaging procedures such as hematoxylin & eosin^7^, high-resolution microendoscopy^7^, immunohistochemistry^8^, or radiomics^9^ that are run in parallel to genetic analysis (e.g. flow cytometry, RNA-Seq, qPCR, western blots). While rich in tissue-level information, these imaging techniques can often overlook heterogeneity that is necessary to describe complex diseases like cancer. At the cellular level, image classification usually relies on brightfield imaging that enables analysis of large fields of view^10^, limiting the single-cell level information necessary to fully describe biological heterogeneity. Continual improvement of automated cell classification frameworks for the diagnosis and characterization of malignancy in cancer remains an unresolved high priority.

The structure and integrity of a cell’s cytoskeletal network has long been known to play an important role in cancer progression. The cytoskeleton is composed of actin microfilaments of globular actin subunits that bind non-muscle myosin II to create actomyosin stress fibers, intermediate filaments (e.g. vimentin, keratin), and microtubules composed of α and β-tubulin. During cancer, this network generally shifts from an ordered and rigid state to an irregular and compliant one to support increased proliferation and motility^11^. More specifically, successful epithelial-to-mesenchymal transitions (EMT) and downstream metastasis are dependent upon reorganization of actin microfilaments via Rho GTPases (Rac1, RhoA, Cdc42), which ultimately guide phenotype^12,13^. Specifically, during EMT, the actin bundles of epithelial cells shift from thin, cortical bundles to thicker, parallel, contractile bundles^14^, allowing for actomyosin contraction and subsequent migration. Thus, some metastatic cancer cells can adopt a similar actomyosin phenotypic profile of healthy migratory mesenchymal cells (e.g. fibroblasts). Yet, metastasis is still more complicated than this overarching model, as some circulating tumor cells (CTCs) do not need to engage in EMT to metastasize. Additionally, CTCs can display a spectrum of both epithelial and mesenchymal biomarkers^15^ and the subsequent intermediate phenotypes^16^. Finally, the cytoskeleton serves as a regulator of gene expression, which can ultimately lead to cell proliferation and activation of various oncogenes^17^.

It is well-known that overall cell shape plays a significant role in its subsequent function^18,19^; one of the best-studied examples is how cells naturally bisect their longest axis during symmetric division. One of our groups has previously shown that cell shape helps orchestrate the mechanochemical signals that direct mesenchymal stem cells to differentiate^19^. Here, we utilized the soft lithographic technique of microcontact printing (μCP) on self-assembled monolayers (SAMs) that was originally described by the Whitesides lab to pattern cells in fixed shapes^20–23^. In essence, the changes in both the actin cytoskeletal structure as well as the overall cell shape can serve as vital markers in cancer diagnostics and progression, potentially improving characterization and diagnosis from automated classification frameworks analyzing heterogeneous cancer cell populations.

In this paper, actin cytoskeletal and morphological data of eight cell lines by high-resolution confocal microscopy provided ample discriminatory data to support an accurate and generalizable classification model. We first quantified single-cell actin cytoskeletal confocal images by defining features based on the spatial configuration and morphology of both cancer and non-cancer cells. We applied supervised feature extraction techniques to identify the cytoskeletal and morphological features with the most discriminating power between cell types. Next, we used support vector machines (SVM) with various kernels to successfully classify cell types in a pairwise approach. Specifically, we applied SVM on all binary cell line combinations to explore classification outcomes between cancer/non-cancer cases against both cancer/cancer and non-cancer/non-cancer cases. Furthermore, certain pairwise comparisons demonstrated improved classification when spatially restricting and normalizing cell shape via μCP on SAMs. Finally, we tested the model’s ability to distinguish a new, dissimilar cell line from a different tissue. This approach confirmed the generalizability of the model to make predictions for completely novel cell lines.

## Results

### Overview of the cell classification framework

**Figure 1:**
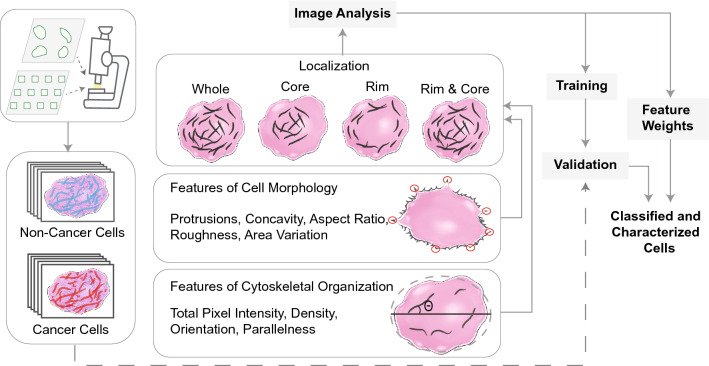
An automated image processing framework quantifies features of cellular cytoskeletal and morphological structure from single cell images. These features were used to train parameters of a classification model and its performance was evaluated using validation data. The algorithm was able to accurately discriminate cancer cells from non-cancer cells and identify individual features that had the greatest influence on classification outcome.

Hundreds of confocal images from eight commonly used cancerous and non-cancerous cell lines were stained for their actin cytoskeletons and collected (Supplementary Figure S1). We spatially segmented cells into various localization classes (whole, rim, core, and rim & core) for analysis (Fig. 1; “Methods”). First, we analyzed the actin cytoskeleton from each image class, quantifying actin fiber intensity, density, orientation, and parallelness (determined by normalized variance of overall fiber angle within the cell). The “whole” image class underwent additional morphological analysis, quantifying shape-based features of the cell: protrusions, concavity, aspect ratio, roughness, and area variance (Supplementary Figure S2). In cancer, malignant, migratory cells are guided by protrusions and parallel contractile actomyosin bundles to help promote efficient cell migration^24^, which often differ from their healthy epithelial precursors. Furthermore, as the cytoskeleton of cancer cells is generally less ordered compared to non-cancer cells^11^, cancer cells often have greater surface area variation known as pleomorphism, which is a hallmark of cancer detection^25,26^. In this way, we could comprehensively describe both the actin distribution and organization at the basal surface as well as the overall morphology of the cell. We took two approaches to validate our model: our *pairwise* approach compared two cell lines in a binary fashion, while our *combinatorial* approach combined multiple cell lines into “cancer” and “non-cancer” categories, later validating novel cell lines to demonstrate generalizability.

### The cell classification model successfully discriminates cancer from non-cancer cells

In order to determine whether we could successfully classify single-cell images by their cytoskeleton and morphology alone, we first sought to train our feature extraction model by quantifying features of interest in a *pairwise* manner amongst eight commonly used human and murine cell lines. Non-cancerous cells included human foreskin fibroblasts (HFF-1), murine fibroblasts (NIH/3T3), and human breast epithelial cells (MCF10A). Cancerous cell lines included human fibrosarcoma (HT-1080), human breast cancer (MDA-MB-231), murine melanoma of moderate (B16–F1) and high (B16–F10) metastatic potential, and human cervical adenocarcinoma (HeLa) cells. Pairwise training identified the optimal hyperplane in an N-dimensional space (N is the number of features) that best discriminates between two cell types. A tenfold cross validation procedure was used for model training and testing.

**Figure 2:**
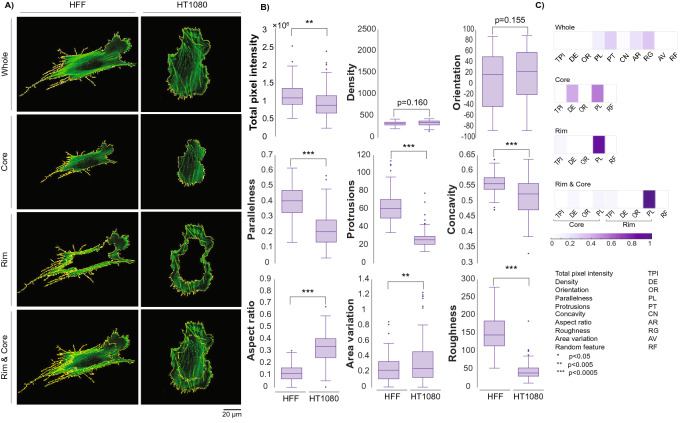
(**A**) Single HFF-1 and HT-1080 cells are segmented based on defined localization classes. (**B**) Features were extracted from the entire data set of HFF-1 s and HT-1080 s images (whole cell, ~ 100 cells per line). Differences between these classes can be observed by eye and by statistically significant differences in features summarizing morphological and organizational properties. (**C**) Fisher discriminant analysis identified the most discriminatory features between these two data sets for all four localization classes.

Within the set of all possible cell–cell comparisons, a physiologically relevant comparison is the HFF-1 against the HT-1080. While the HFF-1 and HT-1080 specifically would not be found in the same tissue, the HFF-1 represents a model human fibroblast, and the HT-1080 represents a model human fibrosarcoma cell—that is, a cancerous cell line derived from similar mesenchymal tissue. Both cell lines are commonly used in cell migration studies^27^. Each raw confocal image was spatially segmented into each of the four primary localization classes, skeletonized for quantification via ImageJ/FIJI, and analyzed to extract features most critical for discrimination (Fig. 2A,B, see “Methods”).

Looking at the whole cell, the non-cancerous HFF-1 displayed a more elongated aspect ratio, increased parallelism, more protrusions, less area variation, greater pixel intensity, and greater roughness than the HT-1080 (Fig. 2B). Yet, statistical significance of quantified features may not necessarily play an equivalent role in classification. An unbiased Fisher Discriminant Analysis identified the combination and contribution of features on classification. Results highlighted the relative impact (weight) of each feature for robust cell discrimination (Fig. 2C). Not surprisingly, when analyzing the whole cell, roughness, protrusion count, aspect ratio, and parallelism of actin stress fibers were most discriminatory. When analyzing other localization classes, different features were highlighted: density and parallelness were most discriminatory for the core class, while parallelness alone was sufficient to discriminate both the rim and rim & core localization classes.

A Support Vector Machine (SVM) was used to perform the classification. SVMs separate classes by solving for the hyperplane that maximizes their distance using various kernels. We employed four common kernels: linear, quadratic, third-order polynomial, and radial basis function (RBF). Looking at the whole cell comparison between HFF-1 s and HT-1080 s, our classification algorithm yielded about 97% accuracy across the four kernels, a promising indication for our methodology.

### Pairwise comparisons show consistently accurate classification

**Figure 3:**
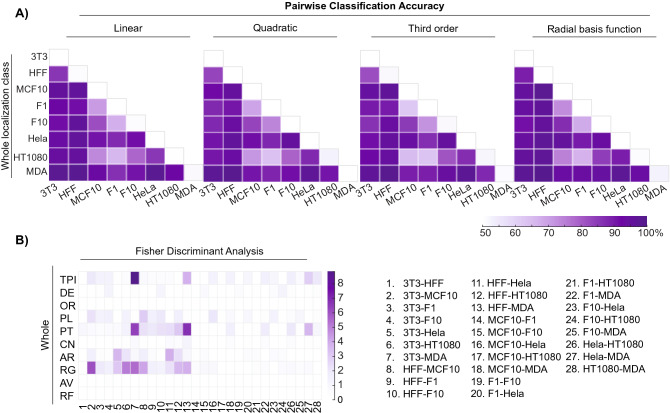
Accuracies of all whole cell pairwise comparisons. (**A**) Heat maps summarize classification accuracies for each SVM kernel; 22 of the 28 pairwise combinations resulted in 90% accuracy and above. (**B**) Fisher Discriminant Analysis reveals the features most important for pairwise discrimination across all 28 comparisons.

We extended the approach to evaluate the remaining pairwise comparisons provided by our data. Accuracy of pairwise classification is outlined in Fig. 3 (whole localization). Here, 22 of the 28 pairwise model training and testing demonstrated accurate classification rates of greater than 90% (other localization classes listed in Supplementary Figure S3). An unbiased Fisher Discriminant Analysis revealed that total actin intensity and stress fiber parallelness along with the morphological protrusion count, aspect ratio, and roughness often played the most significant role in discrimination. However, the latter two features were less relevant when discriminating cancer/cancer pairings.

In addition to the HFF-1/HT-1080 comparison, both the MDA-MB-231/MCF-10A and B16-F1/B-16F10 comparisons are physiologically relevant. The model discriminated between MDA-MB-231 and MCF-10A cells with ~ 94% accuracy but discriminated between B16-F1 and B16-F10 cells with only ~ 74% accuracy. Based on the images acquired via confocal microscopy, it was not surprising that the latter pairwise comparison was more challenging to discriminate; the cells represent the same cancer type from the same species (murine melanoma) and differ only in metastatic potential/aggressiveness^28^. This result marked a true test for our model, as the significant population overlap between these slightly differing phenotypes was difficult to discern. In addition to B16-F1/B16-F10, there were four other pairwise comparisons that were not as accurate (< 85%) as the rest. These comparisons often had similar phenotypes even if the cells came from different species/tissues. For these few weaker pairwise comparisons, a secondary approach to collect and discriminate cell image data was clearly necessary.

### Microcontact printing helps resolve overlapping phenotypes

**Figure 4:**
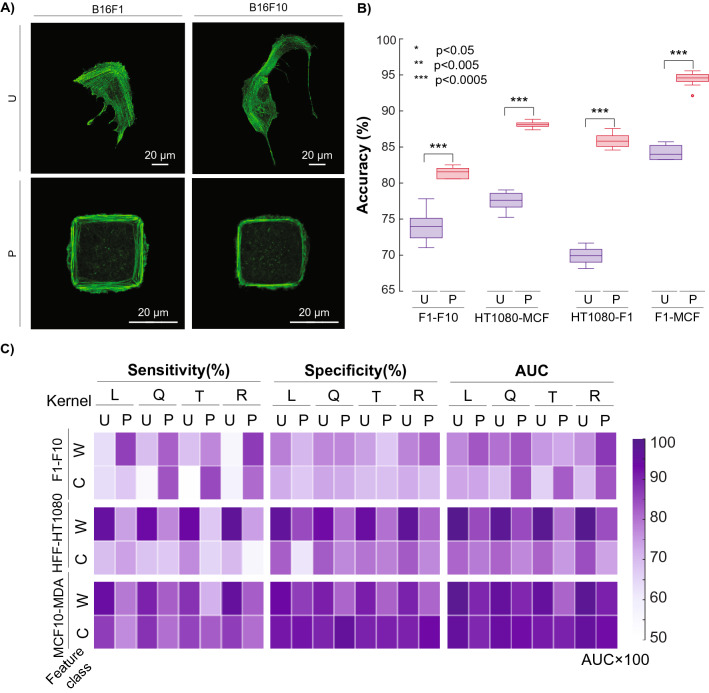
Example of microcontact printing-based image analysis. (**A**) Comparison of B16-F1 and B16-F10 murine melanoma cells when unpatterned (U) or patterned (P) in 900 μm^2^ islands. (**B**) Improvement of pairwise predictive accuracy when hard-to-discriminate cell lines are patterned. (**C**) Sensitivity, Specificity, and AUC of physiologically relevant comparisons B16-F1/B16-F10, HFF-1/HT-1080, and MCF10A/MDA-MB-231 for both patterned (P) and unpatterned (U) cells across both whole (W) and core (C) classes and all four SVM kernels.

In order to improve the predictive accuracy of not only the B16-F1 v. B16-F10 pairwise comparison, but also the other weaker comparisons, we sought to minimize the population overlap between these similar phenotypes by normalizing cell shape. We hypothesized that the phenotypic plasticity seen in cancer^29^ may lead to a wider distribution of phenotypes, potentially confounding discrimination attempts. We posited that normalization of shape could effectively mitigate the spread of phenotypes that made the previous unpatterned cell analysis unsuccessful. Phenotype normalization was done by the microcontact printing (μCP) approach on self-assembled monolayers (SAMs) previously described^21–23,30^. 900 μm^2^ square islands of octadecanethiol (ODT) were stamped on gold-evaporated glass slides and backfilled with tri(ethylene glycol)-terminated alkanethiols to prevent nonspecific protein adsorption. Fibronectin was specifically adsorbed to these ODT islands, which gave the individual cells a platform for attachment and spreading. We found that this area provided cells adequate room to fully spread (i.e. cover the entire square island) in a short amount of time (< 8 h) without allowing the highly proliferative cancer cells to divide. Coupled with nuclear staining, this approach ensured that we analyzed one spread cell per spot (Fig. 4A).

After collecting enough data for each cell type, we re-ran the model with micropatterned (“patterned”) cells. What we found was illuminating: patterning improved the predictive accuracy from 74 to 84% for the B16-F1 v. B16-F10 comparison. In fact, patterning improved the accuracy of classification for three of the least accurate (< 85%) pairwise comparisons (Fig. 4B), demonstrating that shape normalization with methods like μCP are effective in improving image classification in certain situations where native, spread phenotypes are difficult to distinguish. Ultimately, due to the known phenotypic plasticity of cancer, normalizing cell shape may help play a role in effectively analyzing biological properties or monitoring drug response of similar cancer cell types. Overall, 27 of the 28 pairwise comparisons through either unpatterned or patterning methods were able to achieve discriminatory accuracies over 82% with most at or above 90%.

Further quantitative validation can be seen in Fig. 4C, which displays the sensitivity, specificity, and Area Under the Receiver Operating Characteristics (AUC) of the three main physiologically relevant pairwise comparisons. Sensitivity describes the rate at which the model could successfully identify the cancerous (HT-1080 and MDA-MB-231) and more metastatic (B16-F10) cell out of the cancerous/highly metastatic data set. Specificity describes the rate at which the model could successfully identify the non-cancerous (HFF-1 and MCF10A) and less metastatic (B16-F1) cell out of the non-cancerous/less metastatic data set. The AUC curve is a performance metric that quantifies how well our model can discriminate either cancer from non-cancer (HT-1080 & MDA-MB-231 v. HFF-1 & MCF10A, respectively) or less metastatic (B16-F1) from more-metastatic (B16-F10) cells.

We found that for the B16-F1 v. B16-F10 comparison (“whole” localization), sensitivity and AUC are improved as a result of patterning cells in comparison with the same kernel of unpatterned data; however, this was not the case for HFF-1 v. HT-1080 or MCF10A v. MDA-MB-231 comparisons, which were already highly robust when left unpatterned (Fig. 4C). This outcome provided a fundamental conclusion that we found compelling: normalizing cell shape only helped enhance pairwise discrimination in instances where the native, spread phenotypes were highly similar. Initially, we had expected patterning to aid discrimination across most pairwise combinations, but due to the quality of our single cell images, unpatterned cells that were already well discriminated did not require shape normalization.

### Combinatorial approach demonstrates generalizability

**Figure 5:**
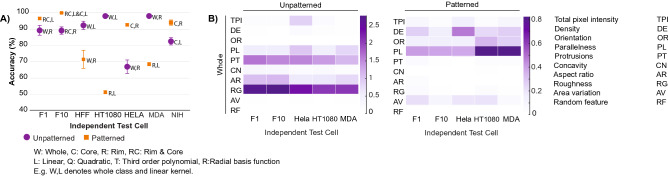
The model demonstrates generalizability towards cancer cells that were not used in model training. (**A**) Maximum predictive accuracy for both unpatterned and patterned cell lines by localization class and kernel. (**B**) Fisher Discriminant Analysis reveals the most discriminatory features for both unpatterned and patterned cells for the whole localization class.

Finally, we evaluated the generalizability of this model on both patterned and unpatterned cells. In this approach, we pooled all five cancer cell lines as a bulk “cancer” class and the two mesenchymal non-cancer cell lines as a “non-cancer” class, as epithelial cancer cells generally adopt a more migratory, mesenchymal phenotype than their epithelial non-cancer precursors (in the case of the HT-1080, it is already derived from mesenchymal cells). Thus, the MCF10A cell line was withheld from model training. As the number of cancer cells was greater than the number of non-cancer cells, the training set was balanced by random sampling from cancer labeled data points to make it equal to the number of non-cancer labeled data points. To evaluate the algorithm’s sensitivity to sampling, we repeated the process (sampling, training, testing) ten times, withholding the same Independent Test Cell, and evaluated (Fig. 5, Supplementary Figure S4A), including leftover images from cell lines utilized in model training (i.e. Consistent Test Cell, Supplementary Figure S4B).

The algorithm was ultimately validated on the independent cell line, demonstrating—in most cases—accurate predictive outcomes. In most cases, some combination of image type (patterned or unpatterned), SVM kernel choice (linear, quadratic, third-order, RBF), and localization class (whole, core, rim, rim & core) yielded a predictive accuracy of 90% or greater. Fisher analysis revealed that while discriminating features differed by cell type, all cases made use of stress fiber parallelness as the most impactful feature. This outcome reinforces the observation that cancer and non-cancer cells have consistent differences in cytoskeletal structure and overall morphology that can be generally predicted by a robust classification framework.

## Discussion

We sought to determine whether differences in cytoskeletal structure and overall cell morphology and organization could provide robust discrimination between cancer and non-cancer cell phenotypes. We utilized feature extraction and machine learning, augmented by SVM, to classify images of single cancer and non-cancer cells. Our single-cell imaging technique provides high resolution, in-depth analysis of cell morphology and cytoskeletal organization. We trained a machine learning model with only 100 images per cell type, demonstrating accurate prediction of dissimilar cell lines across many tissues; every independent test cell achieved an accuracy > 90%. Additionally, we were able to identify key morphological and actin-based cytoskeletal features that were instrumental in discriminating cancer/non-cancer cells across most cell lines. Differences in the density and alignment of the actin cytoskeletal network coupled with differences in overall morphology (roughness, protrusion count, and aspect ratio) of the cell often played the largest role in successful image classification. These differences correlate to many of the known, overarching morphological changes that occur during cancer progression and subsequent EMT^11–14,29^. Ultimately, heterogeneity within and between cell types reinforces the utility of feature extraction models. As advanced imaging techniques that incorporate high, single-cell resolution with increased throughput are made more accessible to both scientists and clinicians, the strengths of unbiased machine learning algorithms in detection, diagnosis, and prognosis of cancer are poised to improve patient outcomes. Additional work has previously been done using convolutional neural networks (CNNs)^31^. While CNNs are a powerful tool often used for image classification of large datasets, interpretability is a known challenge, limiting insight on identifying elements of the cytoskeleton that have the greatest impact on discrimination and their biological interpretation. That being said, both our work and the work of Oei et al*.* highlight the great promise that machine learning portends in cancer classification, and we anticipate that a larger and a more comprehensive single-cell, high resolution data set acquired by a dedicated imaging team would improve model training even further.

Initially, comparing individual cell types allowed us to identify features that could be used as quantitative parameters in both translational and basic cancer research. While the intersection of cell biology and physics is well-studied (e.g. mechanotransduction), most cancer treatments mainly focus on alterations in biochemical signaling. Actin filaments themselves represent a poor drug target due to their ubiquitous role in cardiac, renal, and skeletal tissue homeostasis^32^, but the overall organization of the actin cytoskeleton could be used as a phenotypic guide for drug studies and development^33–35^. Few chemotherapeutic mainstays target microtubules (e.g. taxanes like Paclitaxel), and novel cytoskeletal agents may end up targeting actin-*associated* proteins that show distinct differential expression in tumors (e.g. gelsolin^36–38^). We believe there are many relevant scenarios where such an approach could help investigators and clinicians answer critical questions.

First, this classification framework could interrogate how the actin cytoskeleton and overall morphology of the cell is altered or disrupted. Cancer cells have a distribution of gene expression and downstream phenotypes—it may be informative to discern how cancer cells respond to both existing or new therapeutic agents relative to physiologically relevant healthy cells in the same TME. As a corollary, it may be informative to observe and document consistent phenotypic patterns based on the molecular target within various cancer and non-cancer cells. We speculate that there may be an oncological therapeutic that could remodel the cytoskeleton of a malignant cell (i.e. disordered) to that of a healthy or more benign counterpart. In effect, these sorts of computationally inexpensive analyses could be integrated into traditional biomarker panels that help predict likelihood of therapeutic response. Ultimately, false negatives in oncological screens lower the chance of an early diagnosis with an effective treatment plan and promising prognosis. This powerful generalizability suggests that a similar framework could be useful for developing detailed protocols or kits that can train relevant cell types within a specific TME to ultimately test against a patient biopsy not used in training. It should be noted that the cell lines used in our study are common immortalized lines and are not representative of the types of cells found in primary tumor biopsies; furthermore, our patterning techniques are not representative of the native, 3D tumor microenvironment. Nevertheless, we anticipate that discriminatory cytoskeletal and morphological properties would arise between and within different cancers depending on the origin and grade when similarly cultured on 2D substrates. Significant progress has recently increased the quantitative throughput of pathology at both the tissue-level and single-cell level^39^, which is reflected by the products and services now commercially available (e.g. TissueGnostics, Spatial Transcriptomics, ACD Bio), suggesting that additional pathology techniques to help discriminate between healthy and cancerous cell types will become more routine.

This classification model has the capacity to be extended (both in series and in parallel) to the other cytoskeletal elements that have been observed to be altered in cancer. Not only does this include microtubules, which are targeted by some cancer agents like taxanes, but also includes intermediate filaments, actin-associated proteins, and even the nucleus. While the nucleus would require a different workflow that does not highlight filamentous structures, it is known to face morphological alterations like size, aspect ratio, shape, and number per cell^40,41^. In addition to new phenotypic elements potentially captured by high-resolution confocal microscopy, this type of model could be extended to primary healthy and cancerous cells found within the TME across various biopsy methods. Since fine needle syringe extraction typically yields cell counts on the order of millions^42^, building up a robust model on the order of hundreds of cells would be quite achievable for a team of clinical technicians. Finally, we utilized a microscale patterning technique to normalize cell shape. With the recent advancement of various nano-scale patterning techniques, direct control of subcellular cytoskeletal components on a two-dimensional surface is now more feasible than ever before^43^. These nano-sized adhesive cues guide subsequent cell response, allowing researchers to potentially uncover designs with even better discriminatory power. Ultimately, reliably controlling cell shape in three dimensions in a high-throughput manner is a holy grail in the field of in vitro cell microenvironments, and the continued development of such may greatly facilitate further analysis of native tumor biopsies.

## Methods

### Cell culture

All cell lines were acquired from American Type Culture Collection (ATCC) and cultured at 37C at 5% CO2. Non-cancerous murine NIH/3T3 fibroblast cells were cultured in Dulbecco’s Modified Eagle Medium (DMEM) with 10% bovine calf serum (BCS) and 1% Penicillin/Streptomycin (Thermo Fisher Scientific). Non-cancerous human MCA10A breast epithelial cells were cultured in mammary epithelial cell growth medium (MEBM) supplanted with 4 μL/mL bovine pituitary extract, 1 μL/mL human epidermal growth factor, 1 μL/mL insulin, 1 μL/mL hydrocortisone (all from Lonza), and 100 ng/mL cholera toxin (Sigma-Aldrich). Human foreskin fibroblasts (HFF-1 s), B16-F1, B16F-10 murine melanoma cells, HeLa human adenocarcinoma cells, HT-1080 human fibrosarcoma cells, and MDA-MB-231 human breast adenocarcinoma cells were cultured in Dulbecco’s Modified Eagle Medium (DMEM) with 10% fetal bovine serum (FBS) and 1% Penicillin Streptomycin (Thermo Fisher Scientific). All cell lines were routinely subcultured every 1–3 days to avoid over-confluence and potential cellular quiescence.

### Preparation of monolayers and microcontact printing

No. 1.5 glass coverslips (Fisher Scientific) were sonicated in ethanol, then water, then ethanol for 30 min per cycle before being dried with nitrogen gas to clean the surface. Titanium (50 Å) and then gold (200 Å) were evaporated onto these coverslips using an electron beam evaporator (Thermionics) at 0.2 and 0.5 nm/s, respectively, at 10^–6^ Torr. The patterned surface of polydimethylsiloxane (PDMS) stamps (previously fabricated in-house (30)) were spotted with 10 mM octadecanethiol (ODT) (Sigma Aldrich) in ethanol and allowed to air dry for at least 10 min. Further drying via nitrogen gas was performed to ensure the entire stamp surface was completely dry. Stamps were very carefully placed face-down onto the cut gold-evaporated coverslip with a flat, 15 g weight for 45–50 s before being briefly washed with ethanol, water, and then ethanol. This yielded selective thiol-gold semi-covalent bonding of hydrophobic ODT on the gold slide in the shape of the pattern used. Then, slides were placed in 10 mM triethylene glycol mono-11-mercaptoundecyl ether (Sigma-Aldrich) in ethanol overnight at 4C to backfill the unpatterned regions with hydrophilic alkylthiol. The following day, slides were washed in ethanol, dried with a nitrogen stream, and placed in a 6-well plate (Fisher Scientific) in 1 mL of PBS. Here, fibronectin was directly pipetted into the PBS solution to adsorb onto the hydrophobic ODT pattern and allowed to sit in a humidified chamber for 1 h at 37C. Wells were triple rinsed with excess PBS, but being careful to never go dry before the third rinse so excess fibronectin did not nonspecifically adsorb to the slide.

### Cell seeding

An 8-well chamber slide with #1.5 glass bottom (Ibidi) had a solution of 25 μg/mL fibronectin (Sigma-Aldrich) in PBS (Sigma-Aldrich) adsorbed to the slide for 1 h at 37C to promote cell attachment and spreading for cytoskeletal analysis. After a triple PBS rinse, 5,000 cells were seeded in each well in culture media and allowed to adhere and spread overnight. For patterned cells on the microcontact printed surfaces, 25,000 cells were seeded in each well and allowed to attach and spread for 6–8 h.

### Immunofluorescence

After either unpatterened cells spread overnight or patterned cells spread for 6–8 h, all cells were fixed in 4% paraformaldehyde for 15 min and then permeabilized with 0.3% Triton-X 100 for 5 min at room temperature. Cells were blocked in 1% BSA (Santa Cruz Biotechnologies) with 22 mg/mL glycine for 30 min at room temperature. Then, cells were treated with a 1:40 dilution of AlexaFluor 488 phalloidin (Thermo Fisher Scientific) to label intracellular actin in 1% BSA for 30 min at room temperature with NucBlue nuclear staining (Thermo Fisher Scientific). Cells were triple rinsed with PBS after staining and imaged immediately to ensure high image fidelity. Single cells were taken on a 60X oil immersion objective on a Nikon Ti Eclipse confocal microscope (Nikon Instruments) with the corresponding NIS Elements software. For image acquisition, only the 488 nm (actin) channel was taken, but the 405 nm channel (nucleus) was viewed a priori to ensure that only one cell was in the field of view, which was not always obvious when imaging patterned cells. Laser power was typically between 2–5%. Only the basal layer of the cell was taken, as the underlying cell surface demonstrates the most robust change in cancer cell progression and has the strongest planar F-actin profile. It also allowed us to expedite image acquisition and algorithm design in a reasonable time frame. Images were stored as JPEGs with 2048 × 2048 resolution (0.1 μm/pix/0.03 μm/pix for unpatterned/patterned, respectively).

### Data pre-processing

#### Boundary detection and masking

Single-cell images’ external boundaries were detected and extracted from the background based on the threshold of their pixel values. Pixel values that are larger than the threshold are filtered against the black background with very low pixel value (almost zero). Dilation and erosion, which are fundamental morphological procedures, are used to prevent detection of intracellular areas while the external boundary is detected. The detected external boundaries are plotted on the original image to check the validity of the boundary detection. Each image is masked to make sure the image background, which is not part of the cell and might have some background noise like cell debris, is excluded, so that the analysis is merely performed on the inner cellular region of the image.

### Feature extraction

Features are extracted from each single-cell image within each cell line.

#### *Cytoskeletal features (*Supplementary figure S2*)*

##### Total pixel intensity and density

Total pixel intensity is calculated by the summation of the actin pixel values of each single-cell image. Some cells have more concentrated actin with more robust stress fibers while others, particularly cancer cells, are more diffuse. Actin density is calculated by taking the total actin count and dividing it by the surface area of the spread cell. Pixel lengths are reported from the microscope (e.g. 0.1 μm/pix for 60 × objective image at 2048 × 2048 resolution, 0.03 μm/pix for the patterned images).

##### Directionality and randomness

For directionality, we fitted an ellipse to each cell. Directionality is the average angle of actin stress fibers with respect to the largest diameter of the fitted ellipse. Angles were appropriately weighted based on length of stress fiber, so larger stress fibers contributed more weight to directionality calculation than smaller, more diffuse fibers. Randomness is used to track the overall variation of actin fibers angles. It ranges from 0 to 1 for fully random to fully parallel, respectively. The FIJI LPX plugin was used to calculate the directionality and randomness^44,45^. We developed a framework to make this plugin fully automated.

#### *Morphological features (*Supplementary Figure S2*):*

##### Protrusions and concavity

To calculate protrusion and concavity of each cell, the curvature of the cell boundary is found using MATLAB’s 2D Line Curvature and Normals Package^46^.

1 $$\frac{d\overrightarrow{T}}{dS}= \kappa \overrightarrow{N}$$

where $\overrightarrow{T}$ is unit tangent vector, $dS$ is differential element of border curve of the cell, $\kappa$ is curvature and $\overrightarrow{N}$ is the normal vector. The number of local maxima for curvature of each single cell is stored as “protrusion.” The sign of the curvature indicates convexity/concavity at any point along the cell border. “Concavity” is defined as the number of times the curvature’s sign changes along the boundary, which is further normalized with the total number of points in the boundary.

##### Roughness and aspect ratio

The origin of the coordinate system is coincident with the centroid of the cell and transformed to polar coordinates, which assigns radial values to each point along the perimeter. Roughness, or the presence of non-smooth surfaces, is another indicator of projections in the cell boundary. It is defined as the standard deviation of the radii along the boundary. Aspect ratio is simply the ratio of the radii minimum to maximum along the cell boundary, which provides a normalized measure between 0 and 1. Here, a lower aspect ratio means the cell has a longer, more uniaxial phenotype.

##### Area variation

The standard deviation of spread surface areas of all cells was calculated for each cell line. In general, cancer cells indeed had more variation in phenotype than non-cancer cells.

##### Localization

Synonymous with feature classes, the spatial heterogeneity in actin organization was defined accordingly: The “core” of the cell is the inner 65% of the cell area from the centroid to the boundary. The “rim” constitutes the remaining 35%. The whole cell is further defined as “rim” + “core” or “whole”, which takes the entire cell without summing constituent parts. The latter “whole” classification allows morphological features to be calculated and applied accordingly. These percentages were determined by trial-and-error in order to significantly change the outcomes of classification.

### Feature weights

To investigate the impact of each feature in the classification outcome, Fisher scoring—a supervised feature ranking method—was applied. Features are standardized with their mean and standard deviation before applying the feature ranking and classification algorithm. This was done with MATLAB’s Feature Selection Library^47^.

#### Fisher discriminant analysis

In this algorithm, a projected subspace where the data is well-separated is found by minimizing the within-class variances and maximizing the between-class variances^48^. All data are transformed to the new subspace to explore weights of each feature. Feature weight is the ratio of between-class variances to within-class variances using the following equation:

2 $$\frac{({\mu }_{1}-{\mu }_{2})}{{{STD}_{1}}^{2}+{{STD}_{2}}^{2}}$$

here, ${\mu }_{1}$,${STD}_{1}$, ${\mu }_{2}$ and ${STD}_{2}$ are the means and standard deviations of projected values for two classes (e.g. cancer vs non-cancer). Higher difference of mean with tighter distributions result in higher discriminative power. A random feature with uniform distributions was generated and applied to the feature ranking algorithms as a negative control. The higher weight indicates the more discriminating power, which allows us to rank features for cytoskeletal and morphological characterization.

### Feature classification

We used the Support Vector Machine (SVM) classification algorithm that can identify the optimal hyperplane that maximizes the separation of classes. However, data that was not linearly separable was mapped into a new space that does make the data linearly separable. This allows the SVM to perform an efficient non-linear classification with the so-called “Kernel trick”. We applied four types of common kernels in SVM classification: linear, quadratic, third-order polynomial, and the radial basis function (RBF) kernel. From here, we had two main approaches for classification.

### Pairwise approach

Classification with ten-fold cross validation was performed for all binary combinations of labeled cell lines. For each cell pair, each kernel was used with each feature class (4 × 4). Sampling the data points was repeated ten times and average prediction accuracy values were reported. Prediction accuracy is defined as the number of cell classes predicted correctly to the total number of cells in the test set. For B16F1-B16F10, HFF-HT1080, MCF10A and MDAMB231 sensitivity, specificity and area under the curve (AUC) are also reported. Sensitivity is the number of cases with cancer (HT1080 and MDAMB231) or a more aggressive type of cancer (B16F10) that was predicted correctly out of the total number of known cells in the test set. Specificity is the total number of non-cancerous (HFF and MCF10A) and less aggressive (B16F1) classifications against the total number of non/less aggressive-cancer cells in the test set. AUC is the area under the curve of the Receiver Operating Characteristics (ROC) curve. The ROC curve investigates the performance of the model for all possible classification thresholds. The closer AUC is to 1, the better classification outcome we have.

### Combination approach

Instead of individually labeling cancer cells by their respective cell line, all but one cancer cell line were pooled into one bulk cell line labeled “cancer”. The remaining cancer cell line was left out of the training step to be used as the test set (i.e. “Independent Test Set”). Thus, the test set, unlike the pairwise approach, is made of a cell line entirely not used during training. To balance the larger cancer cell set vs the smaller non-cancer cell set, sample sizes were matched into one comprehensive training set where the number of cancer cells matched the pool of non-cancer cells by randomly selecting a subset of combined cancer cells. The remaining cancer cells were also used in testing (i.e. “Consistent Test Set”). Again, classification was performed on all four kernels and four feature classes. Random sampling was repeated twenty times and the average true positive rates of both the independent test set and consistent test set was reported.

### Statistical analysis

Student’s two-tailed t-test was used for statistical analysis. Pooled and unpooled t-test were used when the variances of two populations are equal and unequal, respectively. An F-test and a Levene test were used to study the homogeneity of the variances when both populations are normally distributed or otherwise, respectively. One-sample Kolmogorov–Smirnov test was used to study whether the data in each sample comes from a standard normal distribution.

## Supplementary Information

Supplementary Information.

## Data Availability

The code and the data that generated and support the findings of this study are available at https://github.com/ZeynabM/cell_classifier. For general correspondence and material requests, please email nbagheri@uw.edu.
