# Supplementary material for: Morphological features of single cells enable accurate automated classification of cancer from non-cancer cell lines
Source: Sci Rep. 2021 Dec 21;11:24375. doi: 10.1038/s41598-021-03813-8 (PMC8692621; doi:10.1038/s41598-021-03813-8)
Supplement: Supplementary file 1 — Supplementary Information. [file 41598_2021_3813_MOESM1_ESM.docx]

**Supplementary Information for**

Morphological Features of Single Cells Enable Accurate Automated Classification of Cancer from Non-Cancer Cell Lines

Zeynab Mousavikhamene^1,4^, Daniel J. Sykora^2,4^, Milan Mrksich^2^*, Neda Bagheri^1,3^*

1. Department of Chemical & Biological Engineering, Northwestern University, 2145 Sheridan Road, Evanston, IL 60208, USA

2. Department of Biomedical Engineering, Northwestern University, 2145 Sheridan Road, Evanston, IL 60208, USA

3. Departments of Biology and Chemical Engineering, University of Washington, 1410 NE Campus Parkway, Seattle, WA 98195, USA

4. These authors contributed equally: Zeynab Mousavikhamene, Daniel J. Sykora

**Corresponding Authors**

* nbagheri@uw.edu

* milan.mrksich@northwestern.edu

| **Cell Line** | **Brief Description** | **Representative Image** | **Cell Line** | **Brief Description** | **Representative Image** |
| --- | --- | --- | --- | --- | --- |
| HFF-1, Human | Non-cancerous fibroblast from foreskin. Typically elongated with aligned stress fibers, may or may not have strong basal fibers in the middle of the cell. Jagged cell shape | 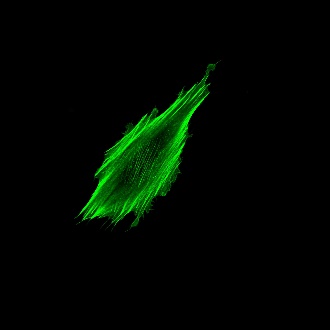 | HT-1080, Human | Cancerous fibrosarcoma cells. Have well-defined stress fibers. Typically less aligned than non-cancerous counterparts. | 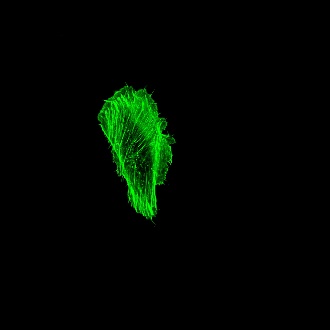 |
| NIH/3T3, Murine | Non-cancerous fibroblasts from Swiss albino mice. Typically elongated with aligned stress fibers. Seem to have slightly more pronounced basal stress fibers in the middle of the cell. | 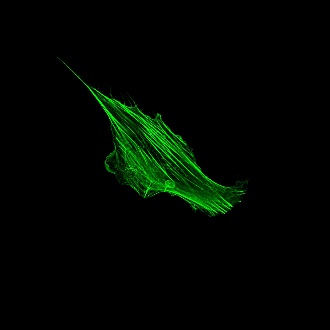 | B16-F1, Murine | Melanoma cells, medium metastatic potential. Mix of spindle shaped and epithelial shaped cells. Large area variation. May have stress fibers within the interior, most often do not. | 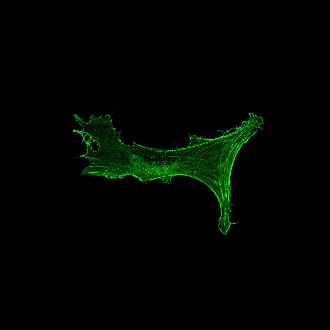 |
| MCF10A, Human | Non-cancerous breast epithelial cells. Less elongated than fibroblasts but still display thick stress fibers. Often have hairy filopodia protruding from the exterior. Generally have a more rigid cell shape. | 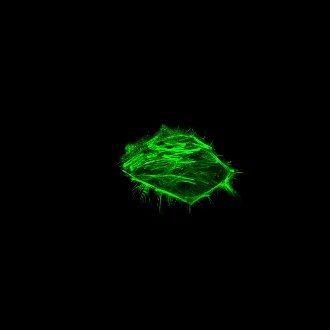 | B16-F10, Murine | Melanoma cells, high metastatic potential. Mix of spindle shaped and epithelial shaped cells. Large area variation. Very few stress fibers in the interior of the cell. | 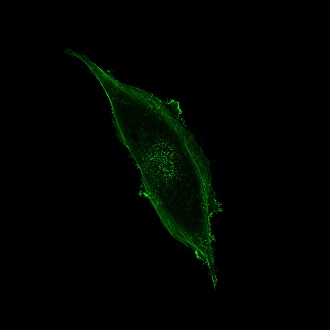 |
| MDA-MB-231, Human | Triple negative basal breast cancer cells. Typically, smaller than other cells with sparse aligned stress fibers interior to the cell; typically have ruffled lamellipodia. | 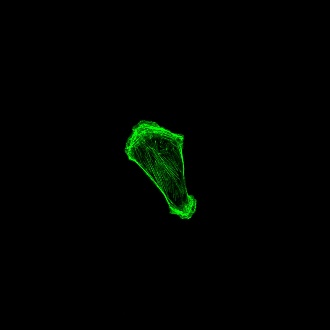 | HeLa, Human | Cervical adenocarcinoma cells. Epithelial cells when confluent/as islands but can appear elongated and mesenchymal when single cell. Have aligned stress fibers with rounded cell shape. Typically fewer defined fibers in the middle of the cell. | 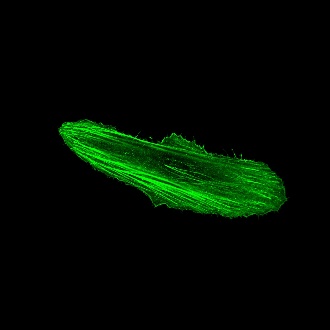 |

**Fig. S1 | Description of Cell Lines Used in this Manuscript.**


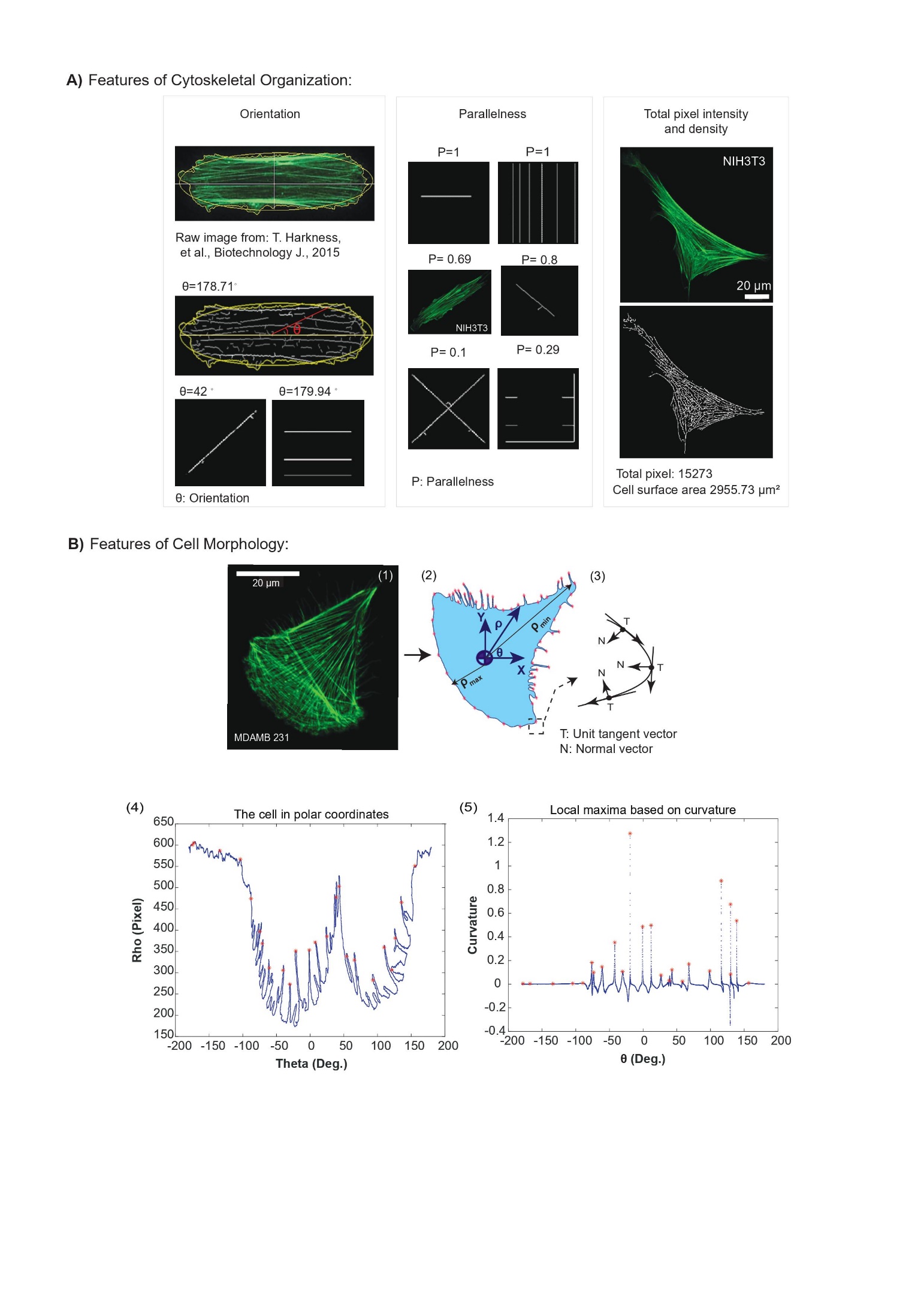


Fig. S2 | Quantified features for each single cell image. A, Features of single cell cytoskeletal organization including orientation, parallelness, and total actin intensity & density are calculated (46). B, Features of single cell morphological features are calculated from the (B1) initial image. B2 is the cell with the transferred boundary to the centroid. An example of minimum and maximum radii (ρ_min_ and ρ_max_) used for the “area variation” feature is presented. B3 illustrates an element of the border curve: unit tangent and normal vectors used to calculate protrusions and concavities. B4 represents the cell in polar coordinates. B5 illustrates local maxima of the corresponding protrusions in B2 (red asterisks). These steps are taken for each individual single cell image.


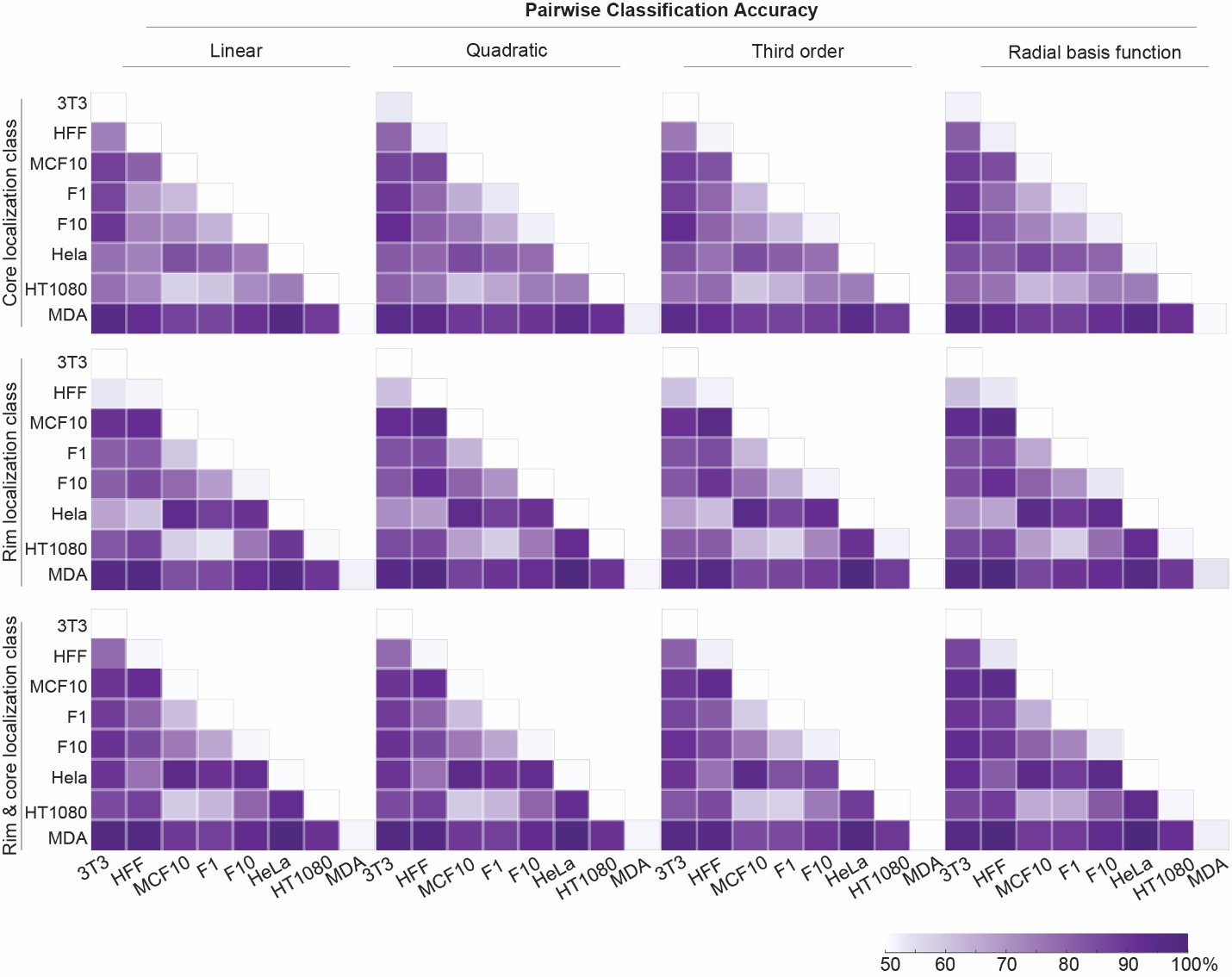


Fig. S3 | Pairwise accuracies for other localization classes. Here, accuracies of all core, rim, and rim & core cell pairwise comparisons are reported. Heat maps summarize classification accuracies for each SVM kernel.


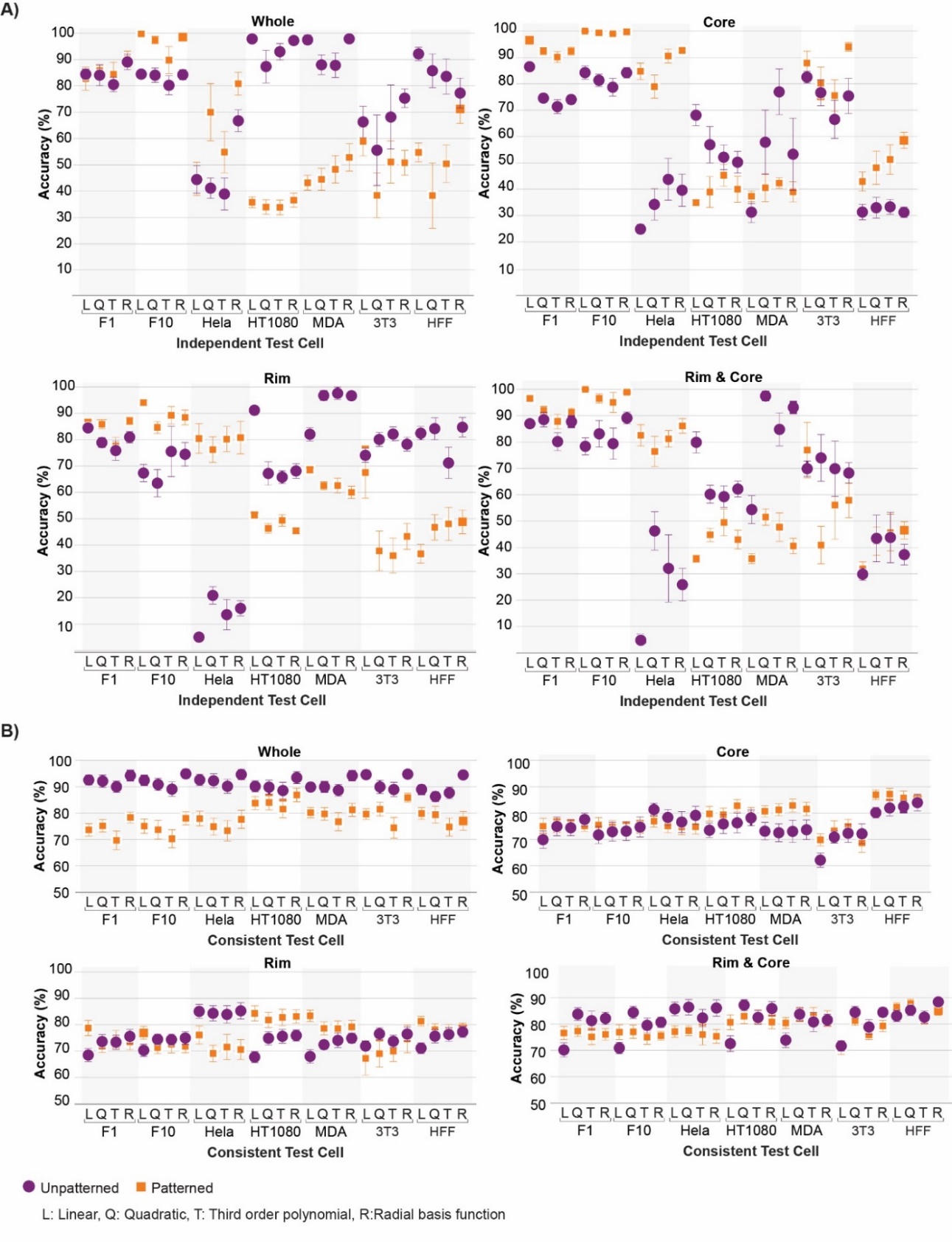


**Fig. S4 | Combination approach accuracies for other localization classes. A,** Accuracies of all core, rim, and rim & core cell Independent Test Sets for both unpatterned and patterned cells are reported. **B,** Accuracies for all localization classes of untested cell data for cell lines included in model training (i.e. Consistent Test Set).
